# A survey of case studies on the use of forensic three-dimensional printing in England and Wales

**DOI:** 10.1007/s00414-022-02872-4

**Published:** 2022-08-08

**Authors:** D. Errickson, R. M. Carew, A. J. Collings, M. J. P. Biggs, P. Haig, H. O’Hora, N. Marsh, J. Roberts

**Affiliations:** 1 Cranfield Forensic Institute, Cranfield University, College Road, Bedford, MK43 0AL UK; 2 School of Life Sciences, Coventry University, Priory Street, Coventry, CV1 5FB UK; 3 School of Chemical and Physical Sciences, Keele University, Stafordshire, ST5 5BG UK; 4 East Midlands Forensic Pathology Unit, University of Leicester, Robert Kilpatrick Building, Leicester, LE2 7LX UK; 5 South West Forensics Imaging Hub, Police HQ, Exeter, Middlemoor UK; 6 Metropolitan Police, 109 Lambeth Road, London, SE1 7LP UK; 7 School of Biological and Environmental Sciences, Liverpool John Moores University, Exchange Station, Liverpool, L2 2QP UK

**Keywords:** Forensic science, 3D Imaging, Blunt force trauma, Dismemberment, Visual evidence, Human remains

## Abstract

3D printing has rapidly developed and been applied in forensic science due to its use in creating demonstrations for courts of law. Much of the literature on this specific topic has focused on the use of 3D printed models in academia, the potential influence on a jury, and its use as a long-term documentation process, but with few actual forensic case examples. This paper offers an insight into the development of 3D printing in forensic practice and how 3D printing is currently being used in the criminal justice system in England and Wales.

A series of case reports were gathered from multiple police forces and forensic practitioners in the UK to identify how 3D printing was being used. These discussions established who was requesting 3D printed exhibits, what type of technologies were being utilised, what type of exhibits were being printed, and resulting feedback for the use of 3D printed material within a criminal case. As a result, this research demonstrates the current use of 3D printing in England and Wales, discussing the associated cases that have been known to incorporate 3D prints. Likewise, this work explores the limitations that have been encountered by forensic practitioners and identifies a series of research questions that should be considered in future investigations.

## Introduction

Three-dimensional (3D) printed reconstructions of forensically relevant materials offer new and broad applications in forensic investigations. To produce a 3D printed model, an initial process that entails the capture of 3D data must be achieved. This methodology has been well described within the literature [1, 2] and has even at times instigated the creation of sub-disciplines, such as virtual anthropology, a term first described by Weber (2015). Although the capture of three-dimensional data is now well instilled within many of the disciplines associated with forensic science, the use of 3D printing is relatively new. The integration of 3D techniques and expertise in the criminal justice system requires specific consideration [3].

There are several publications that review 3D printing while detailing its advantages and limitations in the broad spectrum of forensic science [1, 2, 4]. In addition, there are a handful of experimental studies that relate to the use of 3D printing within forensic investigations [5–8]. These examples include the use of synthetic models as demonstrative evidence within courts of law [9], as a visual example portraying spatial relationships [10], a method for long-term documentation, and the effects of 3D printed models on non-specialists in court, such as members of the jury and legal experts [11, 12]. However, with the exception of using 3D imaging and printing in a medical capacity, many of the examples within the literature are not derived from real case instances and it is important to have data derived from both casework and empirical research to develop forensic evidence bases [13].

One police force in England and Wales is recognised to be using 3D prints in forensic investigations through a collaboration with an academic partner [9]. However, this type of partnership was thought not to be reflective of wider police use. The authors of this article were further conscious that much of the work that has been achieved in academia and research around 3D printing was conducted without input from forensic practitioners. Thus, the applicability of academic research to the needs and experiences of nationwide practitioners was unknown. Our research therefore sought to create a link between research and practice, and to evaluate the status quo of 3D printing in forensic science, towards enabling future research that is informed from practice. As a result, a series of conversations were started between several police forces and forensic providers in England and Wales.

Carew and Errickson [1] presented a set of key future research questions including Who is generating forensic 3D printed models currently, why, how, and where? And how are those 3D prints being used currently with the forensic science framework? To address these questions, a questionnaire was generated to explore how, where, and why 3D prints are currently being used by police and forensic practitioners in England and Wales, and what infrastructure and legislation currently exists to facilitate their practice. The responses to this questionnaire are presented here and it is our intention that by generating this data, the academic community can draw upon common themes, good practice, issues and/or restrictions to inform future guidance and standard operating procedures that can complement existing practices and capabilities.

## Materials and methods

Ethical approval for this study was acquired from Cranfield Forensic Institute within Cranfield University (CURES/13021/2021). To explore the current use of 3D printing within forensic investigations in England and Wales, a questionnaire was designed to elucidate details of cases where 3D printing had been utilised. The questionnaire was sent to various police forces and practitioners operating within England and Wales using convenience sampling beginning with contacts known to the authors. Three individuals representing three police forces did not respond. The questionnaire consisted of eight different sections comprising up to five open-text questions in each section, totalling 19 questions (Table 1).

**Table 1:** An overview of the questionnaire design with three overall topics divided into eight sections providing *n* = 19 questions overall

Topic	Section	Details	Question
Technical details	1. Background details	Case details	1
		Material/evidence type	2
	2. Imaging details	Type of scanner	3
		Software used	4
	3. Printing details	Type of printer	5
		Type of material, colour, etc	6
		Layer height, time to print, removal of support structures, etc	7
		Size of print (e.g., 1:1)	8
		How many printed? (e.g., 1 per juror)	9
Case studies	4. Application details	Presented in court? By who?	10
		Conviction successful?	11
		Supported with booklet/images/photos?	12
Legislation and further considerations	5. Rationale	Who asked for print? And why?	13
		Why this scanning technique? (e.g., access to material or training completed)	14
		Why this printer?	15
		Why this material or colour print?	16
	6. Legislation	What legislation did you consider and why?	17
	7. Limitations/problems faced		18
	8. Any other comments		19

The first section of the questionnaire requested a case summary, details regarding the material and type of evidence printed, the imaging and printing protocols used, and the courtroom application (i.e., whether the case went to court, whether the print was presented to the jury and in what format [alongside supplementary materials for example], and whether a conviction occurred). Additional to these protocol-based questions, the questionnaire also included questions relating to the rationale behind the 3D print requests (which party made the request and why, reasoning behind imaging modality and printer choice, and any discussions on printer material or colour) and legislative considerations (what legislation was encountered or considered, and any legislative issues raised). Lastly, respondents were offered the opportunity to raise any further comments on their experience implementing 3D printing in their cases. These questions were not intended as a rigorous qualitative survey but were designed to provide a method for obtaining consistent background information surrounding the 3D print cases and baseline information from a novel area.

All case data was provided anonymised (no names or dates were included) to prevent identification of the individuals involved. Questionnaire data was collated in an Excel spreadsheet and the data and images stored securely.

## Results

Responses were obtained from two national police forces, one cross-district forensic unit that covers four further police forces, one forensic pathology imaging unit, and one forensic practitioner, providing a combined total of 19 case studies. Three further national police forces were unable to respond*.*

Two of these case studies were omitted from analyses due to their non-association with forensic science since they were archaeological examples, thus resulting in *n* = 17 case studies. It should be noted that some of the case details have been omitted from this article due to their sensitive nature or to protect the identity of individuals associated with the case and the deceased. Additionally, reporting of one of the cases did not follow the format of the questionnaire, but were provided viva voce wherein the technical details were not provided. Thus, the number of full cases was *n* = 16.

### Technical details

**Fig. 1:**
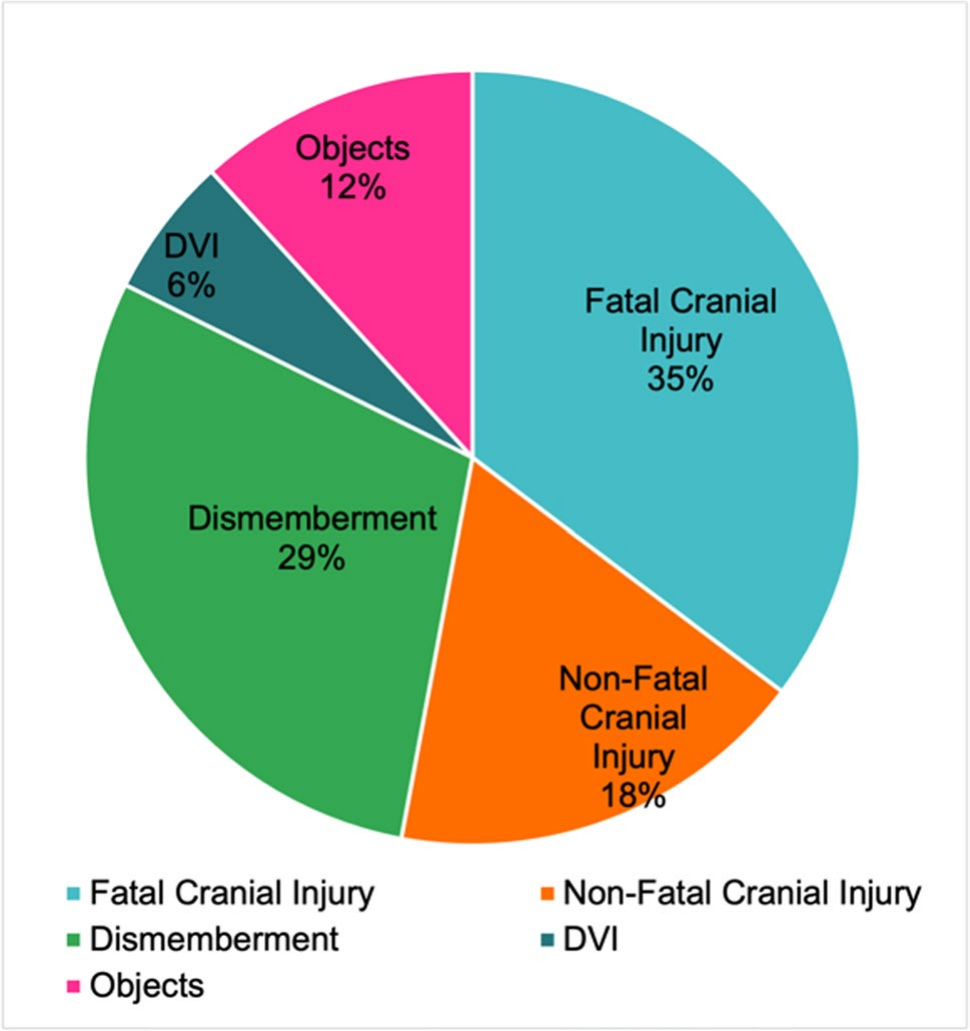
Chart illustrating the types of cases recorded (*n* = 17)

The 17 cases recorded were divided into five categories or ‘types’ of cases based on the nature of the case (question 1). These types of cases were fatal cranial injury, non-fatal cranial injury, dismemberment, disaster victim identification (DVI), and objects. Most cases were fatal cranial injuries (*n* = 6), followed by dismemberment (*n* = 5) (illustrated in Fig. 1).

**Fig. 2:**
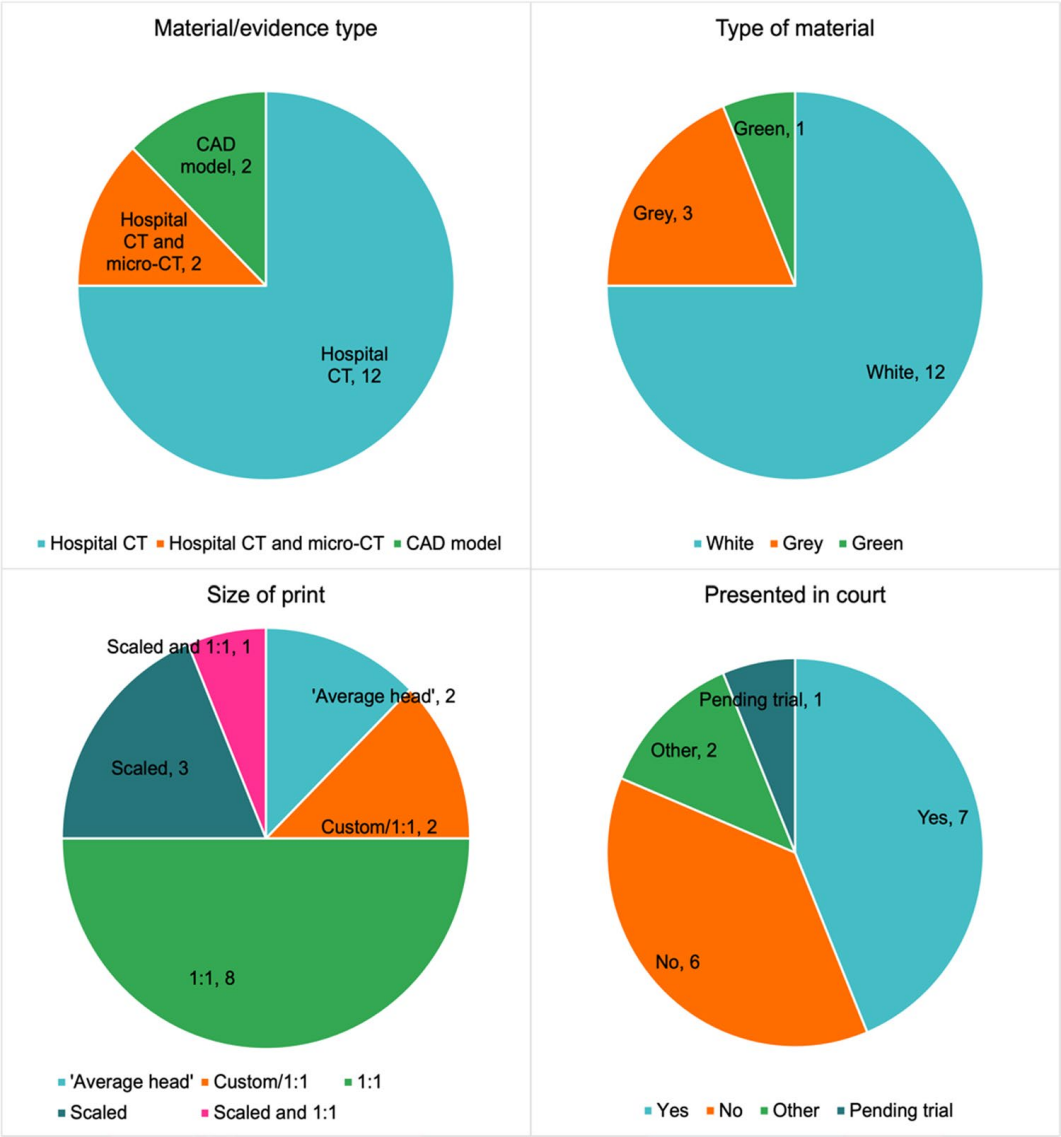
Clockwise from top right, charts illustrating the count from *n* = 16 cases for: the type of print material used; whether the print was presented in court as a visual aid; the size of the print (1:1 ratio); the material/evidence types

The type of material/evidence used in the cases was recorded (question 2) as illustrated in Fig. 2. For most cases (12 of the 16 cases), the object or body parts were imaged using a hospital or clinical multi-slice computed tomography (CT) scanner and/or the CT data was obtained from the hospital in the form of DICOM (Digital Imaging and Communications in Medicine) data. Two of the cases used hospital CT scans in addition to high resolution micro-CT scans (using a Nikon Metrology XTH 225) to obtain a greater level of detail of small pieces of bone in the dismemberment cases. Finally, two of the cases recorded used a CAD (computer-aided design) model. One of these (case 4) consisted of a sphere designed to replicate the circumference of the head of a child, and another was a CT scan from another individual that was designed to reflect the dimensions of a missing individual.

Although the manufacturer of the CT equipment varied (from a Toshiba to a Canon Aquilion), the choice of apparatus was limited to the availability of the local scanner. Following digital data capture, various software packages were utilised to prepare the data for 3D printing (question 4). This is a multi-software process combining two or three stages: initial model generation, post-processing (optional), and print preparation. Where data was provided for the initial model generation, the following DICOM viewers were used to create STL models from raw CT data, OsiriX (10/14), InVesalius (3/14), and Amira (1/14). For post-processing, respondents used either Blender (11/15) or Cinema 4D (4/15) and for print preparation either Preform (Formlabs) (9/16), Cura (Ultimaker) (6/16) or PrusaSlicer (1/16). The micro-CT scan data was viewed using the Nikon Metrology proprietary software, VGStudioMax, and the subsequent 3D models were post-processed using MeshLab.

Similarly, the type of printer utilised was often down to availability and what had been purchased by the company or partner University. As a result, printer type (question 5) included a Formlabs Form 2 (stereolithography, SLA printer), and several fused deposition modelling (FDM) printers (Ultimaker S5, Prusa i3, and a WASP 40/70 INDUSTRIAL). In all cases, the material used was either a polylactic acid (PLA) or a photopolymer in the form of a resin (question 6). All printed replicas were kept to a neutral white, grey, or silver colour, except for one instance where green filament was used (question 6), and all were created to a layer height of 0.2 mm or less (question 7) (see Fig. 2).

The time taken to print the replicas varied depending on the number of prints needed or the number of parts per print (question 7). In these cases, the shortest print time was of an air weapon projectile that took 1 h, and the longest print time was regarding a dismemberment case that included 19 separate parts, resulting in approximately 33 h of printing time. A second factor affecting print time was the size of the replica being produced (see Fig. 2). In most of these cases, the print was either at a 1:1 scale (*n* = 8), or at a reduced scale ranging from 70 to 85% (*n* = 3) (question 8); additionally, case 14 was produced at a 1:1 scale in addition to smaller sections of the cranium being printed at 70% scale. Two cases were recorded as ‘custom/1:1’ (see Fig. 2), in one of these cases (case 3), the 3D print was thickened due to the thin cross-section of the skull needing to be enlarged to ensure that it would successfully print. The other ‘custom/1:1’ case was the custom CAD sphere (case 4) that was printed to be the same circumference of the head of a child. In twelve of the cases, only one single 3D printed replica was produced, and in four of the cases, two were produced (question 9). The second print was produced as an extra replica to be retained by the imaging specialist.

Figure 2 illustrates whether the printed replicas were used in a court of law or not (question 10). At the time of writing, seven of the cases have been through a court of law, six have not, and one is still pending a trial date. Two cases were recorded as ‘other’, in one of these cases the “3D print was used to prove or disprove an allegation” and in another, the print was used for identification of the deceased. Four of the cases that went to court resulted in a conviction, one did not, and in one case, the outcome is unknown (question 11). For the cases where the court details are known and were taken through a courtroom trial, the 3D print was supported with other materials such as an expert witness statement or a booklet containing technical details regarding print production and further 3D images or photographs of the prints (question 12).

### Rationale

Findings from questions 13–19 are discussed below by per case type; however, in all these instances, the senior investigating officer or the forensic expert (forensic pathologist) made the request for a 3D printed model to be used.

### Case study details

#### Fatal cranial injuries

**Fig. 3:**
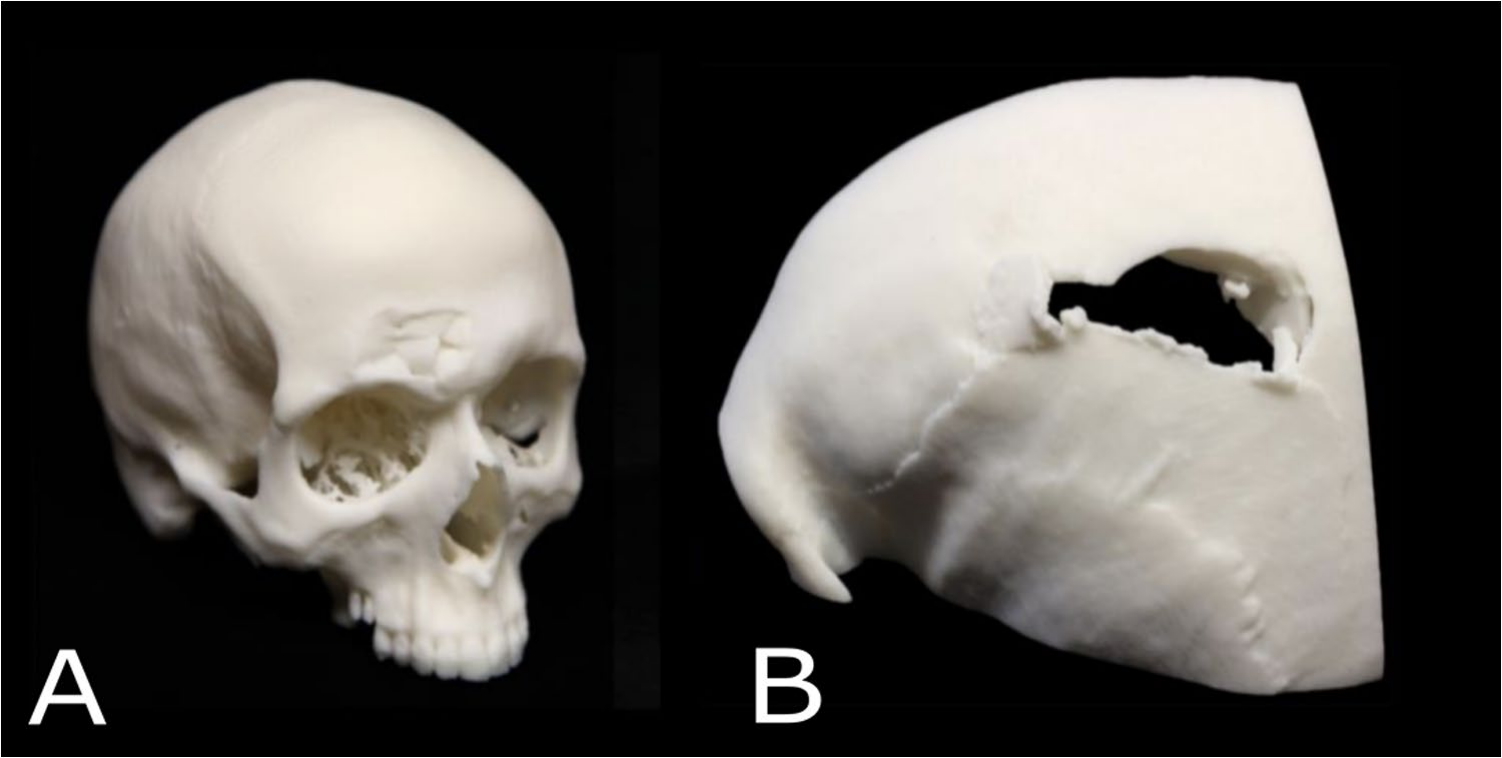
Two examples of 3D printed replicas from cases of fatal cranial injury (A) depression fracture to the right supraorbital region (B) fracture void on the left parietal bone

**Fig. 4:**
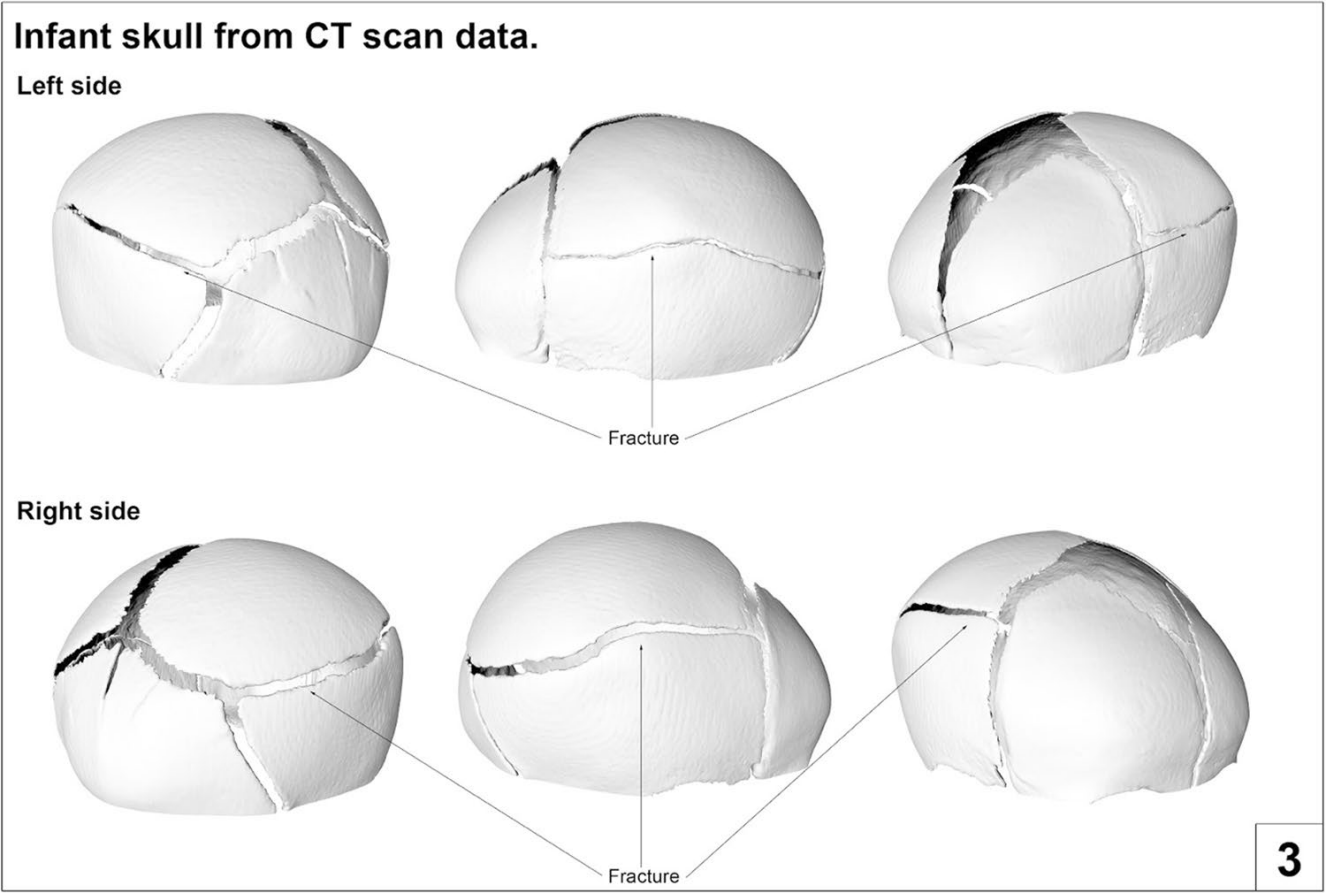
Image taken from a supporting booklet showing different views of a rendered 3D model of an infant cranium exhibiting parietal fracture on both sides

Six cases (cases 1–6) were described where the 3D printed example was a replica of a cranial injury. In all these cases, the 3D printed models replicated fractures on the cranium that had been sustained by blunt and sharp force trauma. In four of these cases, the request for a 3D printed model was made by the prosecution, specifically the senior investigating officer and was presented in court by the medical expert, namely the pathologist. In the remaining two cases, either the case was dropped, or a guilty plea was given prior to the trial, thus the printed replicas were no longer needed. Out of the four cases that went to court, three cases used a single 3D print. The fourth case utilised two copies of the same 3D print; one copy was held by the pathologist as they gave testimony from the witness box, while the other copy was passed around the jury. Figures 3 and 4 provide examples.

Supporting information was always provided for those cases that went to court. This either took the form of a booklet that incorporated images of 3D renders taken using Cinema 4D, anatomical illustrations of the injuries together with photographs of the 3D print, or a statement explaining technical details with accompanying images to provide visual continuity for the jury members.

In all of these (*n* = 6) cases, the 3D print was used as a visual tool to show the injuries sustained. The printed models were a combination of 1:1 replicas of the full cranium or cropped to show a specific region of interest. This varied depending on whether the internal surface was of interest in the visualisation of injuries, or whether the build volume of the 3D printer could fit the entire object.

#### Non-fatal cranial injuries

**Fig. 5:**
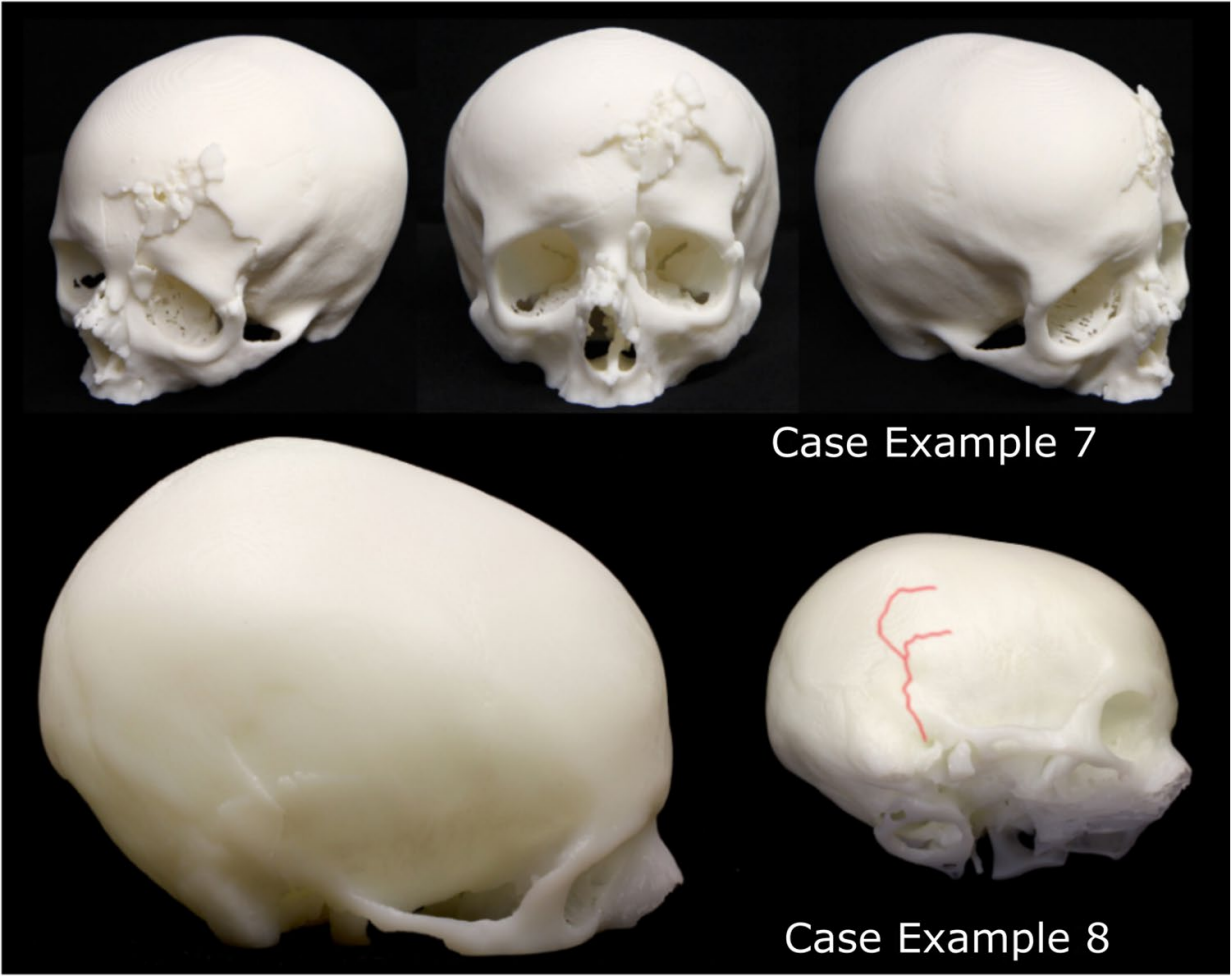
3D printed model examples from two non-fatal cranial injury cases. Case example 7: depression fractures to the frontal bone and nasal region in an adult. Case example 8, fracture spanning the right temporal and parietal bones (highlighted in red) in an adult.

Three of the cases that created 3D printed models were examples of non-fatal cranial injuries where the victims survived (cases 7–9). In these cases, the investigating officer was influential in requesting the model and in one of these cases, the request was supported by the prosecuting barrister. However, the 3D printed model was only taken into court for one of these cases. Fig. 5 provides example models from two cases.

In the first non-fatal cranial injury case (case 7), an adult female was assaulted wherein she sustained blunt force cranial trauma. CT scanning had been undertaken as an emergency assessment prior to medical intervention, and an enquiry was later received from the Senior Investigating Officer as to whether this clinical scan could be used to produce a model displaying the skull injuries. However, after inspecting the physical print, the prosecution team decided not to introduce it into the court trial, considering that it might be deemed excessive alongside sufficient alternate evidence that had already been gathered. An expert injury opinion had previously been provided by a forensic pathologist who had conducted an examination of the victim while in hospital. This forensic pathologist had not seen the CT scan, and the opinion had been based solely on external examination findings. The 3D printed model was subsequently shown to the same pathologist, who was able to provide an additional statement commenting in detail upon the underlying skull fractures that had not been visible externally. A separate statement was produced to explain the technical details of model production and to allow for visual continuity with the model. The case resulted in a conviction for attempted murder.

In the second case (case 8), a young adult female was assaulted and sustained blunt force trauma to the cranium. There was no opportunity to obtain police photographs of the external injuries as due to the severity of the injuries, lifesaving treatment had to be undertaken quickly. However, a clinical CT scan was available due to the emergency assessment that had been carried out and 3D printed models could be produced and presented in court by the forensic pathologist. A scaled-down replica of the whole skull was used to explain the total fracture distribution, while a separate 1:1 skull segment was used to demonstrate the internal and external features of a depressed fracture element that bore size and shape details indicative of the weapon used. This was supported by a statement detailing the model production process, and the trial resulted in a conviction for attempted murder.

**Fig. 6:**
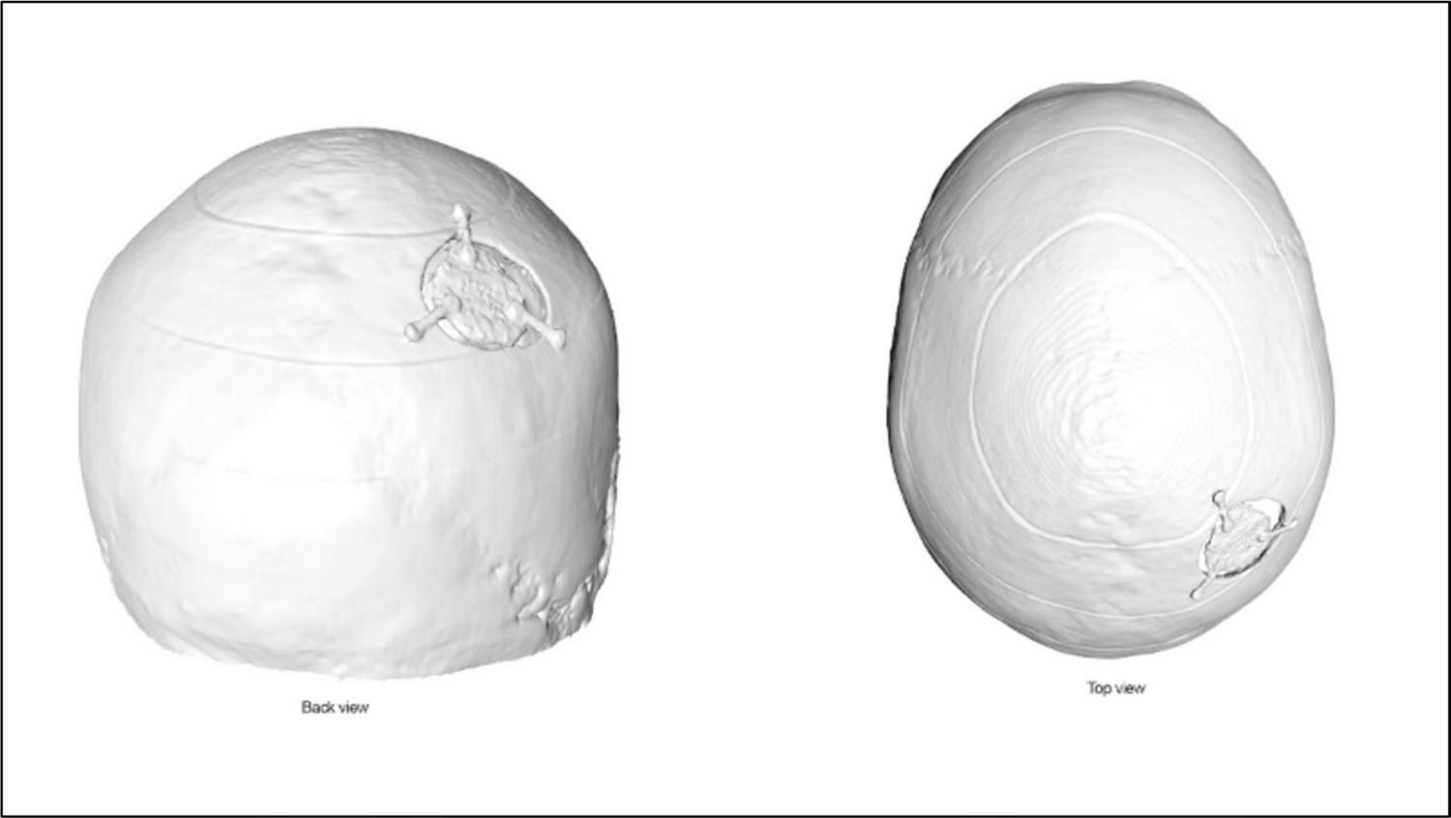
Image taken from a supporting booklet from case 9: the post-surgery 3D model can be seen prior to printing

In the third case (case 9), the 3D printed model was used to show the damage sustained to the skull after serious grievous bodily harm. Pre-surgery scans were initially going to be used; however, the scans did not display correctly as the data looked distorted with poor resolution. Therefore, post-surgery scans were utilised, and medical experts were consulted to ensure the print and supporting material were represented accurately. The print was subsequently used in court by the medical experts for the prosecution, supported by a booklet that displayed anatomical illustrations and 3D renders of the skull (Fig. 6).

#### Dismemberment cases

Five of the reported cases that incorporated 3D printing involved dismembered or disarticulated remains (cases 10–14).

**Fig. 7:**
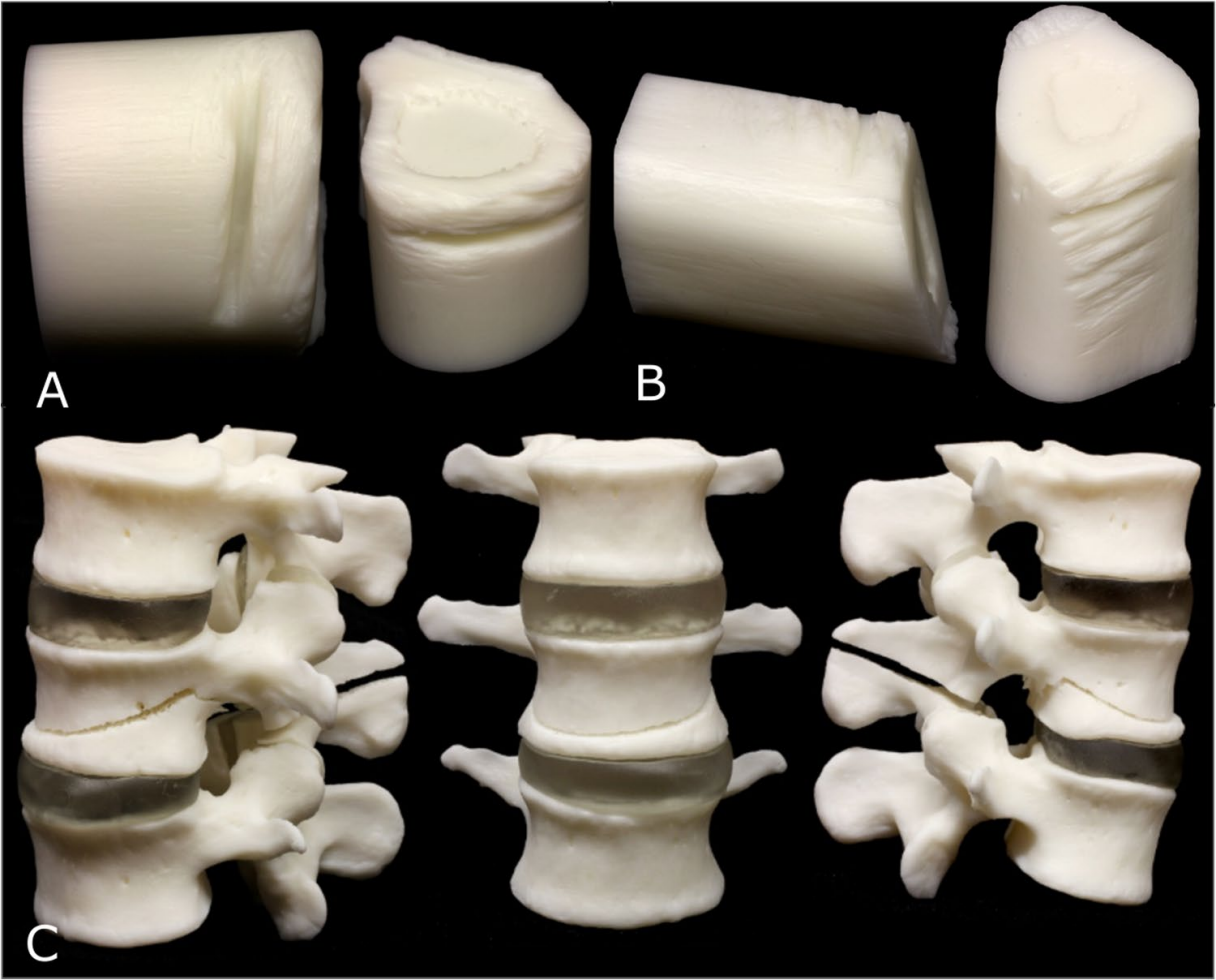
A series of 3D prints examples from case 10, a dismemberment case where the partially dismembered bone samples were 3D printed. (**A**) Print of false start kerf, (**B**) A micro-CT model of the humerus dismemberment site with numerous shallow kerf marks (**C**) A series of 3 images of the three, 3D printed, partially dismembered lumbar vertebrae from oblique (left), frontal (middle), and lateral (right) views

In the first dismemberment case (case 10), the remains of a dismembered adult male in varying states of decomposition were found across a number of sites. Post-mortem CT scanning was conducted on each of the recovered body parts and micro-CT was undertaken on the de-fleshed areas where the acts of dismemberment could be observed. Due to the severity of the injuries, the investigating officer requested the use of 3D printed models to be used in court to help demonstrate the actions performed during the dismemberment (Fig. 7). In this example, a trial was not required because the defendant plead guilty. However, the 3D printed models were able to support the actions described by the suspect detailed in their statement. The high-resolution micro-CT scan also offered greater visibility of the injury details.

**Fig. 8:**
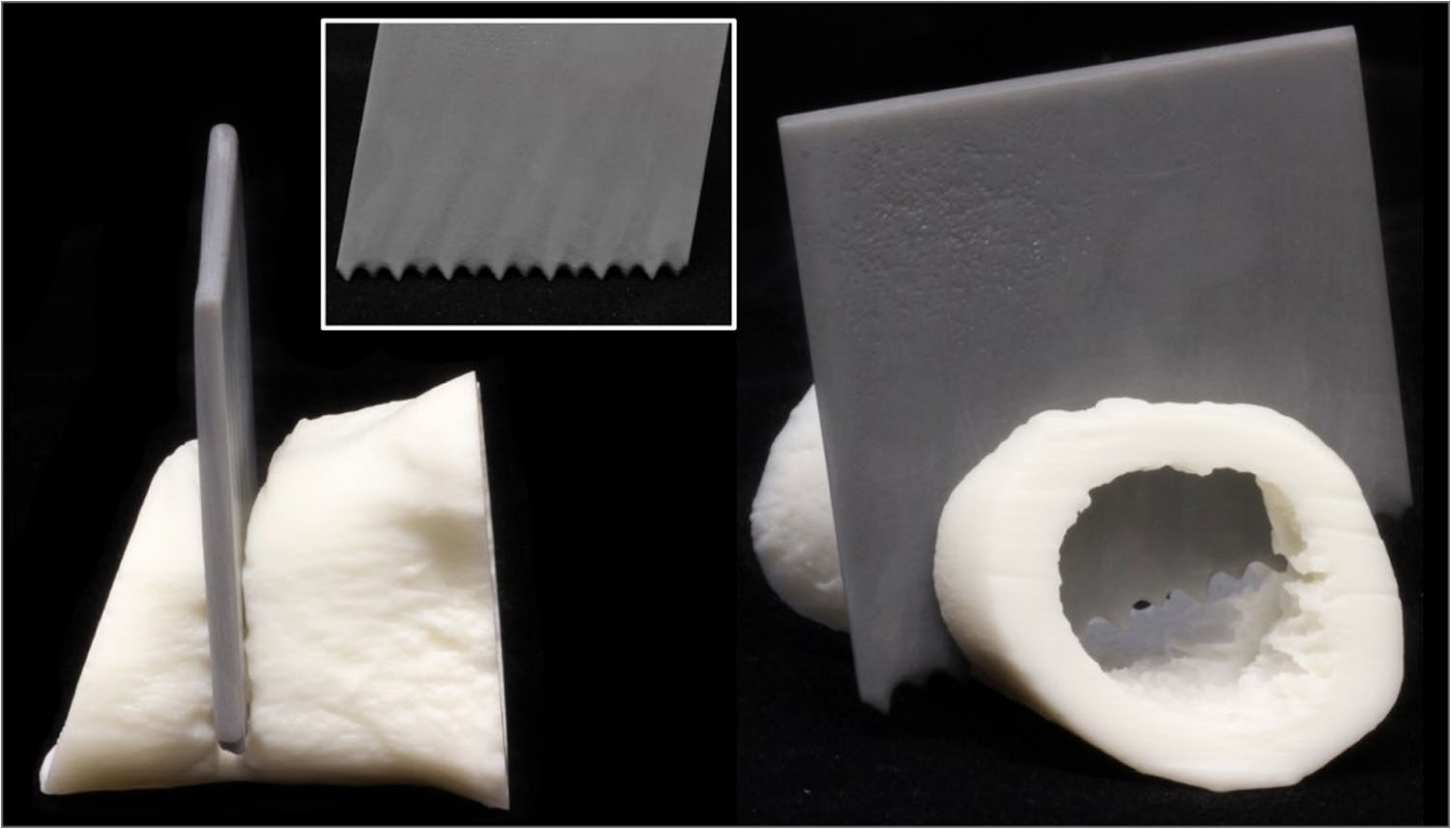
Case 11, a dismemberment case where partially dismembered bone samples and the saw blade were 3D printed. Left, a 3D print of near complete false start kerf with blade print inserted into the voided area, and right, a cross-sectional view, (Insert) tooth pattern of blade demonstrated in 3D print

In the second dismemberment case (case 11), an adult male was murdered, dismembered and the body was concealed in a clandestine grave in a woodland area. Like the first example of dismemberment, post-mortem CT and micro-CT were both utilised. Importantly, in this case a suspect had been identified, and a tool potentially used for the dismemberment was found in their possession. Consequently, the investigating officer sought to demonstrate the fit of the recovered saw blade against the saw marks on the bone to the courtroom. Thus, a portion of the bone was 3D printed (in a white colour) that contained an almost complete ‘false start kerf’ and a completed cut, as well as a replica of the saw blade (in a darker grey colour) (Fig. 8). This case demonstrates a novel example of an interactive visual model that was used to help the jury to understand key events from the crime.

The third dismemberment case (case 12) discussed an adult female who was missing and presumed to have been murdered. A suspect was charged, and a small freezer was recovered from their premises with the working theory being that the victim had been dismembered and stored within the domestic freezer. No human remains were recovered, but the investigating officer wanted to ascertain whether it was possible to store a dismembered female of a similar size to the missing individual in the freezer given its known dimensions. Forensic imaging specialists used laser scanning to digitally capture the domestic freezer and determine the internal volume dimensions. Given that the body of the missing female had not been recovered, a 3D CT model of an individual of estimated similar proportions was sourced from within a clinical research database. Smoothing was applied to the surfaces of the scans to remove any potential identifying features and the 3D model was subsequently ‘virtually dismembered’ along the most common lines of dismemberment (according to the expertise of the experienced forensic pathologist).

**Fig. 9:**
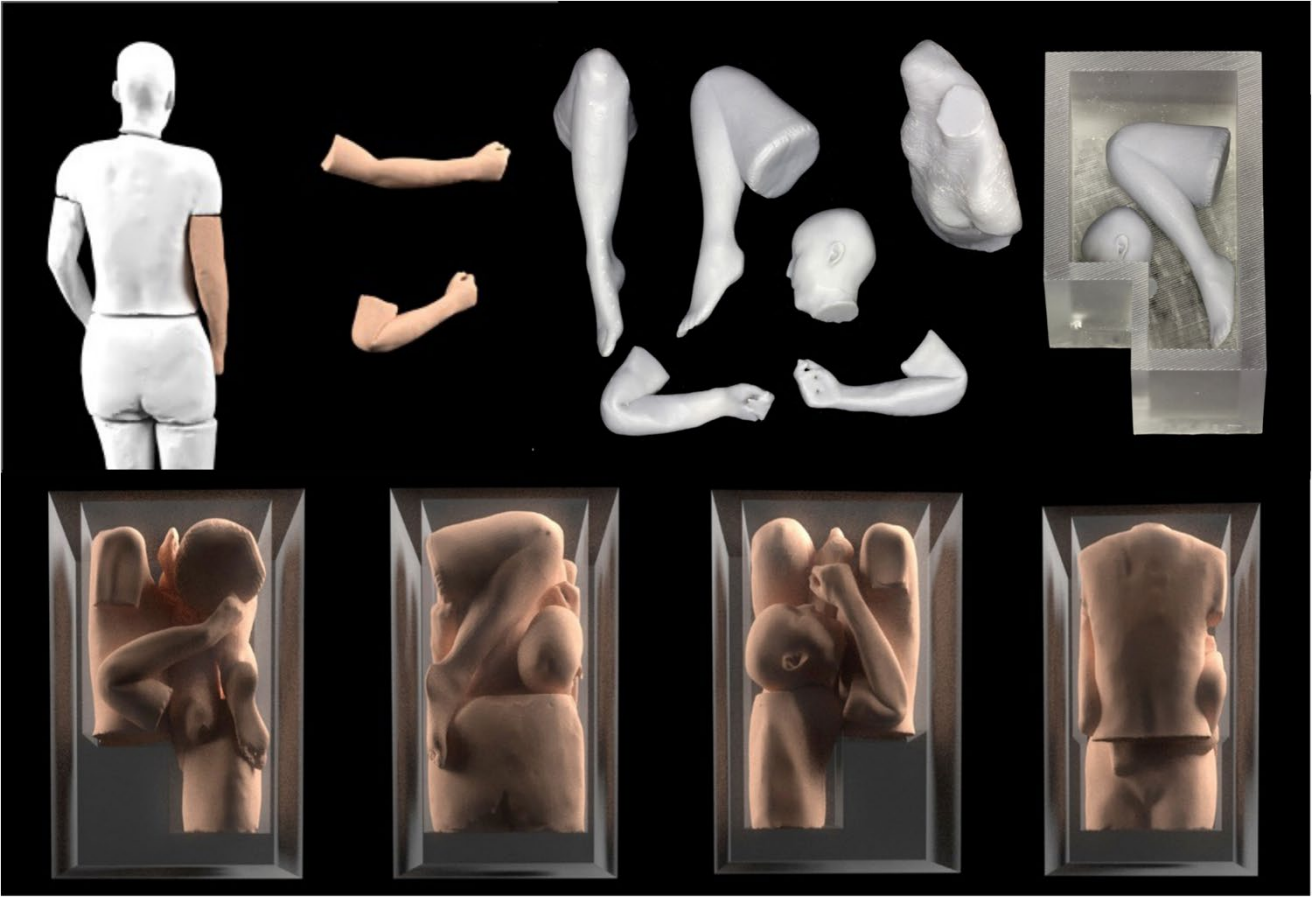
Virtual and 3D printed replicas of the freezer and pseudo-dismembered body, demonstrating the theoretical fit of the components in such a space

Each individual part of the pseudo-dismembered model was then 3D printed as a miniature version (9% scale of the original), to allow for an optimum arrangement of parts within a scaled down space representing that of the freezer. None of the 3D printed models in this dismemberment case were used in court. Once the best arrangement of packing was identified using the 3D printed replicas, this was recreated virtually so that a simulation could be used in court to show that the body of an individual of this size and shape could, in theory, have fitted inside the recovered freezer if dismembered (Fig. 9).

The fourth dismemberment case (case 13) centred around a suitcase containing human remains. The suitcase was CT scanned prior to removal of the body parts and the subsequent post-mortem examination. Several days later, another suitcase was found also containing human remains. After examination, all remains were confirmed as belonging to the same individual using DNA analysis, while part of the left shoulder and proximal shaft of the left humerus remained missing. Several days later, possible human remains excavated from a third location were examined by a forensic anthropologist. These remains were identified as being part of a left scapula that was articulated with a proximal humerus, encased in burnt soft tissue. The forensic anthropologist also identified a blade injury in the exposed humeral head. This body part was sent for CT scanning at the same hospital where the previous post-mortem examinations had been undertaken, and initial confirmation that this body part belonged to the dismembered individual from the suitcases was achieved by virtual reconstruction of the CT scans. Further analysis of the blade injury, using traditional casting and microscopy methods, was undertaken by tool marks expert in a forensic laboratory. A DNA sample was also taken from the shoulder, which returned a positive identification to the other body parts.

In this example (case 13), the humerus with the blade injury was further documented using micro-CT; however, the resolution of the data was considered too poor to assist the case. Furthermore, although the 3D models and subsequent 3D prints were useful for demonstrating to barristers and police officers the case in detail, it was suggested that there was no good scientific reason to perform the micro-CT scan in terms of improving or enhancing the results already obtained by the experienced forensic practitioners.

**Fig. 10:**
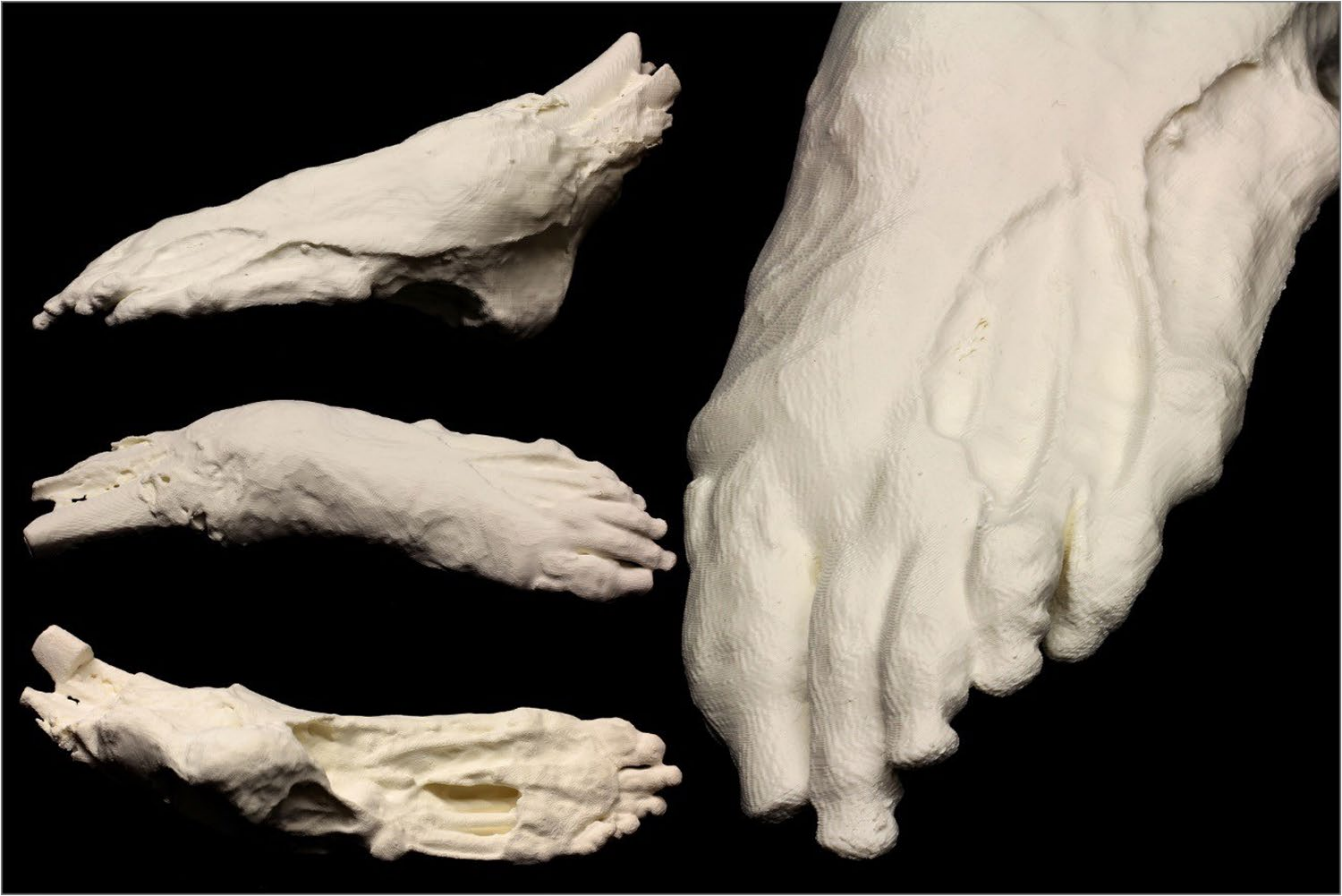
A partially mummified human foot that was documented and 3D printed to ensure the actual human remains were not contaminated or exposed to further decomposition

The final dismemberment case reported (case 14) was not used as an investigative or courtroom aid. This case study focussed on a partially mummified human foot that was found by a dog walker. In this example, the investigating officer did not want to risk repeated exposure and thus degradation of the body part; however, they requested a 3D printed example simply for reference and convenience of handling in an office environment (Fig. 10). The 3D print provided an ethical, sanitised object that was useful for demonstration purposes.

#### Objects

The following two cases (cases 15 and 16) concern objects that were 3D printed within the forensic context.

**Fig. 11:**
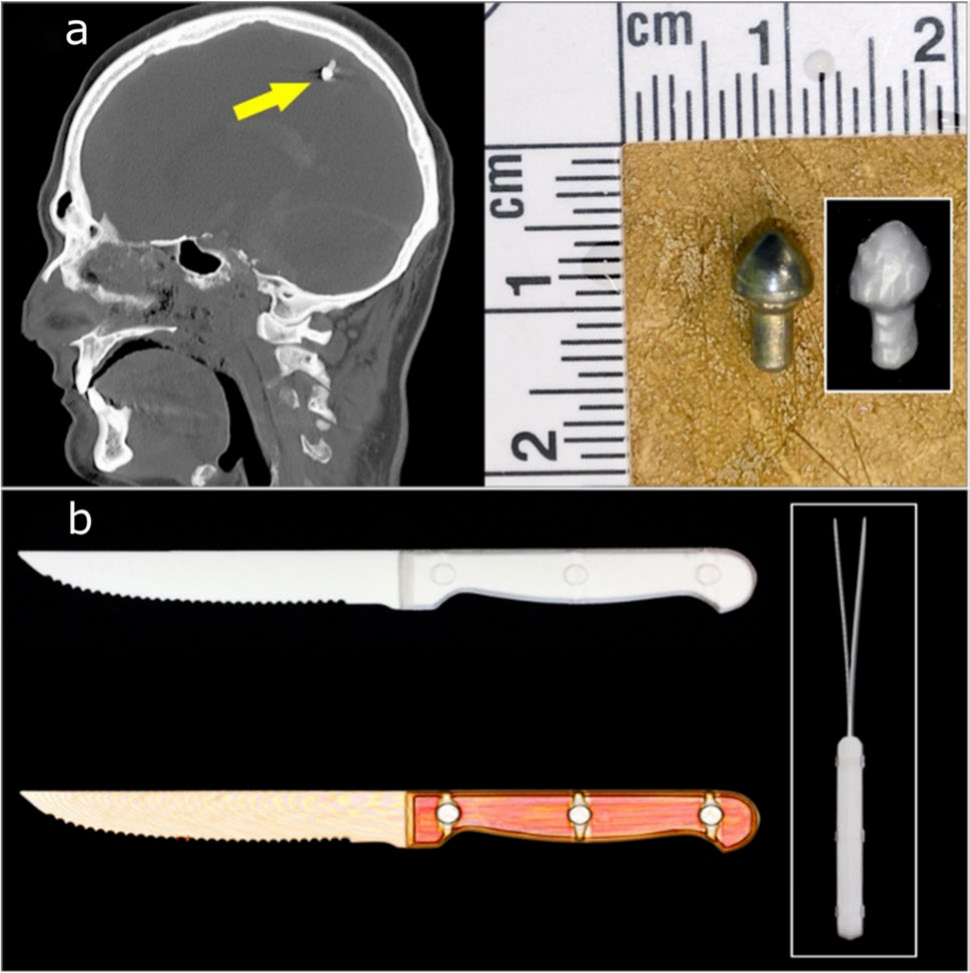
3D printed weapons or artefacts. (**a**) A projectile that was documented in situ and printed prior recovery from the body. (**b**) A knife that was scanned in a sealed container and printed without being removed.

The first case (case 15) followed a self-inflicted air rifle head injury. This example was initially considered by the police to be a possible medical event because no exit wound had been identified and the deceased had been found slumped in a chair with the air rifle lying on the floor next to them. A post-mortem CT scan was conducted as part of the forensic post-mortem examination and a metal artefact was identified in the scan data. In this example a 3D printed replica was not requested by the investigators, but 3D printing was carried out prior to the autopsy to test the viability of recovering virtual ballistic materials. A 1:1 3D printed version was produced before the start of the post-mortem examination, which provided a good impression of the size and shape of the projectile for the forensic pathologist (Fig. 11a).

The second object case reported (case 16) focussed on the homicide of an adult male. The investigating officer wanted to ask the defendant to demonstrate to a jury how the weapon (a knife) was held and what actions were made with it. As the original weapon was sealed as evidence in a container, the investigating officer sought to create a 3D printed model of the knife for the courtroom demonstration. A CT scan of the sealed container was therefore requested, and a 3D printed model produced (Fig. 10b). In addition, a statement was prepared for the court to explain where the knife model came from, and this included technical details of how it was produced and was supported with images for visual continuity. An added advantage of these images was that superimposed views from opposite sides of the knife revealed the residual distortion of the blade, which had been bent during the stabbing incident (Fig. 11b inset).

#### Disaster victim identification

**Fig. 12:**
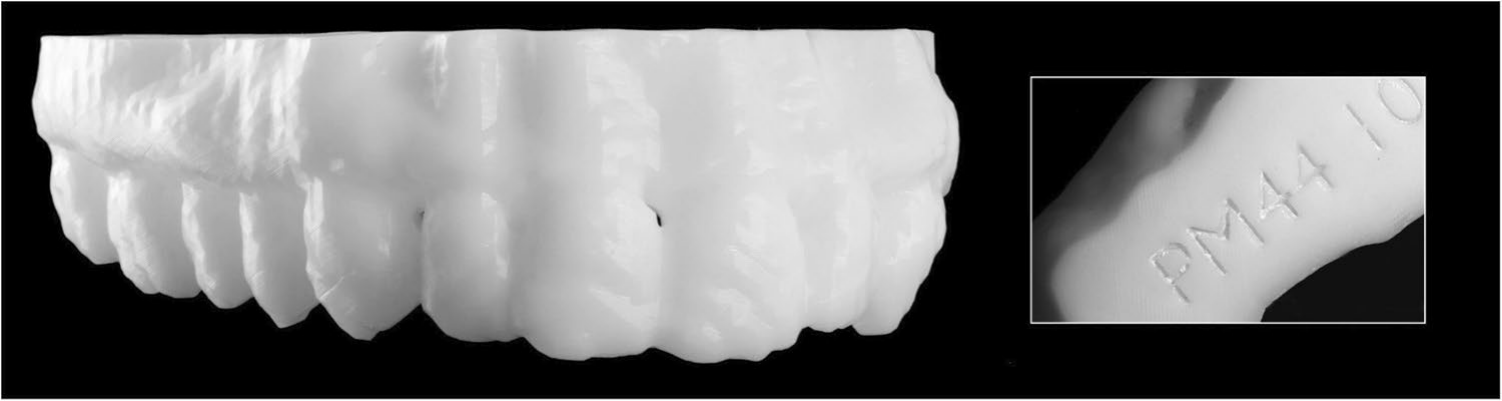
A 3D printed maxilla from a burned individual that was not subjected to further physical alterations. The 3D printed model still allowed for an identification to be made

A final case (case 17) of 3D printing was reported that was not used in court but is a useful example of 3D printing being used in disaster victim identification (DVI) in England and Wales. This example was also reported by Biggs and Marsden [14], wherein a young adult male was one of multiple burned fatalities from a building explosion, that also included a severe fire and collapse of the premises. A CT scan was performed as per DVI procedures and as a non-invasive alternative to physical examination. HM Coroner had not authorised invasive examinations due to the non-suspicious nature of the incident, and so disfiguring the facial region of the deceased with incisions was to be avoided if possible. A CT scan of the cranium was instead used to facilitate dental examination and identification of the deceased, in line with DVI recommendations such as in the INTERPOL Guidelines [15]. In this case, the 3D printed model was used by the forensic odontologist to establish a positive identification by comparison to ante-mortem dental records, to the satisfaction of HM Coroner (see Biggs and Marsden [14] for further details) (Fig. 12).

### Legislation

For all cases reported here, no specific legislation was considered or consulted with regards to the generation or admissibility of the 3D reconstructions (question 17). In three of the cases, however, the medical experts and investigating officers were consulted on the final print to ensure that accurate representation of the injuries was depicted. In one of the cases, ethical concerns around identifiability were considered since the printer was positioned in a staff-only shared space.

### Limitations and further comments

A range of limitations were provided in response to the questionnaire (question 18). Several of these were case-specific whereas others were more generalised limitations associated with the virtual imaging and 3D printing process. Case-specific limitations were observed in cases 5, 6, and 16. The issues encountered in case 5 related to the young age of the victim. The cranial sutures of the deceased were still open and meant that the segmented bones of the skull were in multiple separate pieces as opposed to one cranial unit. In this instance, a ‘fake’ internal material was generated beneath the bone to allow printing as one piece. There were concerns that it may not be clear to a lay audience that this internal material was a generated addition, and it was felt perhaps with the capacity to do so, printing in two materials would have been of benefit. Both cases 6 and 16 included metallic materials. In case 6, a metallic inclusion within the skull disrupted surface detail and the quality of the CT scan. Case 16 on the other hand included a knife with a metal blade and plastic handle. In this case, despite using the scanner’s SEMAR metal artefact reduction system, the difference in material properties of the blade and handle meant that they were treated as two separate surface files during the segmentation process and fused later.

The generalised limitations associated with the imaging and printing processes included print scaling, timescales, file sizes, and print material quality. In multiple cases (*n* = 6), it was reported that prints could not be printed full size as a single print due to limited printer bed size. Further software limitations would not allow prints to be scaled up, causing a particular issue in case 3 where the bone was particularly thin. Limitations surrounding timescales were noted in case 14, including both the virtual imaging and post processing stages as well as the printing stages. There were concerns around potentially unrealistic timescales to produce prints, especially from complex scans containing multiple tissues, and print times exceeding working hours (many taking over 12 h to complete* n* = 6). In the two cases where micro-CT was implemented (case 8 and 9), limitations were noted regarding file sizes being particularly large and challenging to manipulate. This issue was overcome by cropping out any unnecessary data and printing only the specific region of interest, thus allowing the focus to be maintained on the area for discussion. Finally, print material finish was raised as a concern in one case using ‘early versions of the white and grey resins’ produced a ‘translucent, shiny’ finish, although the respondent does note that later versions of the resins have improved.

Respondents were provided with the opportunity to make any further comments on their experience of using 3D imaging and printing in casework (question 19). For the cases with further comments, many respondents shared the view that 3D imaging and 3D prints were of benefit to the cases, even in case 4 which was subsequently dropped. There was a shared opinion between several cases that the 3D prints were particularly useful for the clarification and contextualisation of injuries and their placements, as well as for demonstrating the size and shape of the weapon in question. It was noted in case 9 that the 3D print allowed an element of interactivity where the jury could remove the printed saw blade from the cut mark to examine both the teeth of the blade and the internal features of the kerf. In case 11, the method of cropping the skull for printing was regarded as advantageous, facilitating the viewing of both the internal and external surface of the injury. Finally, where Blender was used, it was remarked on how comprehensive the platform was as a free software.

## Discussion

3D printed replicas are increasingly being used in forensic casework. This study has been the first to explore how, where, and why 3D printed models are currently being used by forensic practitioners in England and Wales. The results obtained have provided insight into seventeen distinct case examples covering different types of skeletal injuries, and objects associated with forensic investigation. Interestingly, all printing cases involved trauma inflicted to the body, and even if the object printed was not anatomical, it reflected the object that inflicted the injury. However, it is important to note that even though 3D evidence has been made for courtroom purposes, no examples here were used as substantiative evidence to prove theories associated with the cases. The role of 3D printed exhibits in a court of law must not be exaggerated and in fact, all examples here were simply used as visual aids to support expert witness testimony.

It should be noted that case 6 demonstrates a major advantage of 3D imaging and printing. In this example, the pre-surgery CT scan was the only surviving record of the injury prior to medical treatment. This scenario draws on parallels with Woźniak et al. [16] and demonstrates that pre-surgery CT data is extremely valuable as key evidence can be documented before any changes occur. Being able to retrieve data after modification, death, or even reburial can be a valuable resource to a forensic investigation.

The scanning and printing methods used in the reported cases were those available in-house and/or where the medical procedures took place. Given potential issues around printing timescales and increasing workloads, in future, access to appropriate or additional equipment and processes may be required, thus highlighting the need to bridge the gap between forensic science provision, policing and academic institutes. It could be argued that there is the need for experts who specialise in 3D data capture and processing to be involved in the early stages of an investigation. For example, not all procedures and protocols will be the same, and although some materials are straightforward in terms of capture and modelling, some materials can be extremely complicated, for example requiring intricate segmentation or processing. Therefore, an awareness of the steps required, the time scales needed to process data, and the need for trained experts in both anatomy and data processing is important.

It is not surprising that specific legislation was not considered in any of the cases reported here, since there is limited relevant legislation or formal documentation that exists with respect to the use and production of 3D printed courtroom exhibits. The Criminal Procedure Rules (2020) state that experts must help the court by giving an opinion which is unbiased [17], and an accurate 3D printed replica of the point of discussion may be used to demonstrate a fact. However, there is no consensus on the type of case 3D technology should be used in and no standard operating procedures or guidelines for best practice for producing prints. Moreover, there is a lack of substantial supporting research regarding the psychology around bringing 3D visual aids into a courtroom. While there is an increasing body of literature presenting cases where 3D imaging or printing has worked particularly well, such as included in this current work, there remains a communicative void between those involved. Consequently, there is a perceived bias in the literature that presents positive outcomes with little evidence exploring the incidences where there was or has been the potential for the use of 3D aids to be disadvantageous to a case. Furthermore, there lacks a discussion regarding best practice for the storage of 3D prints and the large quantities of associated digital data (raw scans, 3D surface files, g-codes, etc.) pre-, during, and post- trial. Post-trial associated material should be archived with the rest of the case material or returned to the police as exhibits (or a combination of both). However, without legislation or guidelines in place, there is concern that with numerous forensic actors involved in a 3D production [3], no single party holds responsibility for this data and this poses risk of potential ethical and legal issues.

The digital data generated from 3D imaging practices warrants further consideration. Working with large digital files is a challenge, particularly with respect to micro-CT which generates large raw datasets as well as large 3D reconstructions. The resulting data requires adequate computer processing power, digital storage, and consideration for longevity during and following trials. It is also important that those actors requesting the prints are aware of the realistic timescales of creating such work. The process of generating accurate 3D prints is not a simple one and is likely to require a bespoke approach depending on the complexity of the material of interest. Some materials, such as dry bone, are more straightforward as a single material, whereas evidence types containing multiple materials and/or of complex shapes are likely to require different imaging modalities or increased segmentation and post processing approaches, which can therefore result in extended model and print generation times. A further question to consider surrounds the ownership of the 3D data. In traditional osteoarchaeology, it may be assumed that the individual who produced the 3D reconstruction is the owner; however, several actors could be involved in a reconstruction process and there is no clear guidance around digital intellectual property in such frameworks [18]. In the forensic context, this digital data forms part of a casefile and needs to be accounted for as part of a chain of custody. 3D data should be archived accordingly, disclosed to the court, and if requested made available to opposing counsel. Establishing protocols on the storage of the data, the time that data should be stored, the use of the data, and the disposal of data are all important considerations, especially as these datasets include personal identifying data of victims of crime.

A further question worth raising is who should be requesting a print, who should be generating a print, and is a print necessary? Currently, there are no standards to follow, or qualifications required, and a variety of forensic practitioners from different disciplines are creating 3D printed replicas. Given the technical aspects such as CT scan segmentation and 3D model generation, there is a question as to who is qualified to perform these tasks, and for those undertaking such tasks to have recognised competency expertise in that area. Forensic practitioners and medico-legal experts are required to demonstrate their competence and knowledge of the Criminal Justice System in order to practice and act as expert witnesses in the UK. This same standard should apply to those who are producing 3D prints. Furthermore, there is a need for trained and anatomically cognisant experts to perform the segmentation of biological material. The authors agree that a 3D printed reconstruction needs to be used appropriately and in conjunction with evidence from other experts, especially when multiple specialists are involved in a single case (such as forensic anthropologists, pathologists, radiographers, toolmark examiners, etc.). Each expert consulted should also be credited as appropriate. Any future procedures and protocols around forensic 3D printing will need to be adaptable to case-specific challenges and as such, transparent and effective communication between those requesting the 3D print and those responsible for generating the print, is required to ensure that mutual expectations are met. Case 17 provides an interesting discussion on whether the 3D printed model was even necessary. For instance, if the required detail can be examined on the 3D models, should we physically replicate the data for the sake of printing? This is a new consideration, but the question demonstrates the need to ensure the whole process of 3D data capture is considered from the very start.

The discussions underpinning this study have additionally facilitated the establishment of a 3D printing working group within the UK Forensic Capability Network (FCN). The role of this working group is to unite representatives from academia, police, and forensic practice towards developing national guidelines and standardised 3D imaging and printing protocols. It is our opinion that such collaborations are crucial for encouraging genuinely constructive future research and assisting effective and reliable integration of 3D technology into the forensic investigation and courtroom process.

There are a vast number of discussion points and because there are currently no good practice guidelines. From this research, the following list of recommendations towards best practices are suggested.

- ○ Inferences from 3D reconstructions should only be made within areas of expertise.
- ○ Forensic actors need to have an awareness of the limitations of the techniques and of their own expertise.
- ○ Consideration to be given to intellectual property rights and acknowledgement of all actors involved in the case
- ○ Authorisation from all actors involved in a case before a print is presented in a court of law.
- ○ Quality control checks are vital to ensure accurate 3D printed reconstructions.
- ○ Forensic 3D imaging should not be conducted in isolation from the experts that will be/have been analysing the primary evidence.
- ○ There is currently no evidence to support the use of 3D printed replicas for analysis or interpretation purposes.
- ○ 3D prints can be suitable for courtroom visualisation and demonstration purposes.
- ○ Research is needed to compare traditional methods with findings from 3D printed material.
- ○ Further collaborative research between forensic service providers and academic researchers is needed for a holistic approach.
- ○ The evidential value of 3D printing should not be overstated — it is impossible to know if a 3D print helped (or hindered) to secure a conviction in a court case without significant research (such as post-trials surveys).

## Summary and conclusion

This work has shown that 3D printed exhibits are increasingly being requested and used within courts of law. These requests are from a range of forensic practitioners including the police, anatomical specialists and pathologists, for use by the expert witness. Scientific literature often focuses on 3D printing of human remains; however, this article expresses that there are further applications of 3D printing that could be used in the criminal justice system. Similarly, the authors believe that other uses in conjunction with an increase in frequency of 3D printing being sought for criminal trials is imminent. It is clear that the application of 3D printing to the courts in England and Wales is beneficial. For instance, evidence that has since been destroyed, changed due to destructive analysis, or buried (in the case of human remains) can still be replicated and demonstrated to a judge and jury. Likewise, as demonstrated in this discussion, 3D printed replicas can also be utilised to challenge or support a statement of actions.

The findings from this research enable future studies to have practice informed research, facilitating improved applicability and holistic approaches to forensic 3D printing. The discussions had and responses gained from the study presented here have been key in approaching the necessary future research questions as outlined by Carew and Errickson [1], demonstrating that while in its infancy, the use of 3D printed material within courtrooms in England and Wales is becoming a reality. Of the cases explored in this study, there were consistent themes regarding the methods used in print generation, the application and type of cases utilising 3D printing. However, many of these cases are currently unknown to the wider academic community, and the public. It is imperative for the progression and integration of 3D information within the criminal justice system that researchers and practitioners are working towards a shared goal. Furthermore, it is also important that current academic research is aligned in its approach, representing the reality of casework requirements, and ensuring that any future studies associated with 3D printing (such as the reaction by a juror, or judge to printed material), are guided by the actual application.

Although the application of visual material to the legal process can be advantageous, it does not negate the fact that there are currently no standard practice guidelines for the use of 3D printed material within the courts. It is of paramount importance that these guidelines are developed so that the admissibility of evidence is not risked. Currently, there is a concern that future efforts may be hindered by the omission of good practice, and these guidelines should not solely focus on the replica itself, but the initial data capture too. Afterall, the final product reflects the initial scan and its parameters and if these are insufficient then any resulting reconstructions may be compromised, which could ultimately lead to misleading information being presented in a courtroom and unsafe rulings [19].

There must be a global effort towards creating guidelines for better informing the courts on the particulars associated with a 3D printed exhibit. This research has shown that there are valuable methods for doing this through the development of booklets as supporting information that detail the rendering process or how the final models have been created. If these actions can be incorporated, and the cognitive effects of 3D prints on the jury understood further, then the use of printed objects in the court has a promising future.
